# Rituximab use in adult glomerulopathies and its rationale

**DOI:** 10.1590/2175-8239-JBN-2018-0254

**Published:** 2019-12-20

**Authors:** Joana Eugénio Santos, David Fiel, Ricardo Santos, Rita Vicente, Rute Aguiar, Iolanda Santos, Manuel Amoedo, Carlos Pires

**Affiliations:** 1 Hospital Espírito Santo de Évora, Departamento de Nefrologia, Évora, Portugal.

**Keywords:** Rituximab, Glomerulonephritis, Antibodies, Monoclonal, Immunosuppressive Agents, Treatment Outcome, Drug-Related Side Effects and Adverse Reactions, Rituximab, Glomerulonefrite, Anticorpos Monoclonais, Imunossupressores, Resultado do Tratamento, Efeitos Colaterais e Reações Adversas Relacionados a Medicamentos

## Abstract

Glomerulopathies are one of the leading causes of end-stage renal disease. In the last years, clinical research has made significant contributions to the understanding of such conditions. Recently, rituximab (RTX) has appeared as a reasonably safe treatment. The Kidney Disease: Improving Global Outcomes guidelines (KDIGO) recommended RTX only as initial treatment in antineutrophil cytoplasm antibody associated vasculitis (AAV) and in non-responders patients with lupus nephritis (LN), but these guidelines have not been updated since 2012. Nowadays, RTX seems to be at least as effective as other immunosuppressive regimens in idiopathic membranous nephropathy (IMN). In minimal-change disease, (MCD) this drug might allow a long-lasting remission period in steroid-dependent or frequently relapsing patients. Preliminary results support the use of RTX in patients with pure membranous LN and immunoglobulin-mediated membranoproliferative glomerulonephritis (MPGN), but not in patients with class III/IV LN or complement-mediated MPGN. No conclusion can be drawn in idiopathic focal segmental glomerulosclerosis (FSGS) and anti-glomerular basement membrane antibody glomerulonephritis (anti-GBM GN) because studies are small, heterogeneous, and scarce. Lastly, immunosuppression including RTX is not particularly useful in IgA nephropathy. This review presents the general background, outcomes, and safety for RTX treatment in different glomerulopathies. In this regard, we describe randomized controlled trials (RCTs) performed in adults, whenever possible. A literature search was performed using clinicaltrials.gov and PubMed.

## Resumo

As glomerulopatias figuram entre as principais causas de doença renal terminal. Nos últimos anos, a pesquisa clínica efetuou contribuições significativas para a compreensão desse grupo de patologias. Recentemente, o rituximabe (RTX) surgiu como um tratamento razoavelmente seguro. As diretrizes do Kidney Disease: Improving Global Outcomes (KDIGO) recomendam o RTX apenas como tratamento inicial na vasculite associada ao ANCA (VAA) e em pacientes não respondedores com nefrite lúpica (NL), embora não sejam atualizadas desde 2012. Atualmente, o RTX parece ser pelo menos tão eficaz quanto outros esquemas imunossupressores na nefropatia membranosa idiopática (NMI). Na doença por lesão mínima (DLM), o medicamento pode proporcionar um período de remissão duradouro em pacientes córtico-dependentes ou com recidivas frequentes. Resultados preliminares corroboram o uso de RTX em pacientes com NL membranosa pura e glomerulonefrite membranoproliferativa (GNMP) mediada por imunoglobulina, mas não em pacientes com NL classe III/IV ou GNMP mediada por complemento. Os achados a respeito de glomeruloesclerose segmentar e focal (GESF) idiopática e doença por anticorpo antimembrana basal glomerular (anti-MBG) não são conclusivos em função do pequeno número, porte e heterogeneidade dos estudos publicados até o presente momento. Por fim, a imunossupressão com RTX não é particularmente útil na nefropatia por IgA. A presente revisão apresenta o racional da prescrição de RTX nas diferentes glomerulopatias, desfechos e segurança. Nesse sentido, foram incluídos ensaios clínicos randomizados (ECRs) realizados em adultos, sempre que possível. Pesquisas bibliográficas foram realizadas nas bases de dados do clinictrials.gov e no PubMed.

## Introduction

Glomerulopathies are rare diseases^1^^,^ 2, with different presentations, clinical courses, and outcomes^3^. For these reasons, clinical trials are challenging to design or perform.

Some glomerulopathies are considered to result from the invasion of the kidney by immune complexes or by cells of the innate and adaptive immune system^4^.

Current glomerulopathies treatment is based on the use of corticosteroids and immunosuppressive drugs acting in different pathways of the immune system response such as cyclophosphamide, azathioprine, cyclosporine, tacrolimus, and mycophenolate mofetil (MMF)^5^. These drugs are toxic and have many adverse effects. Therefore, new therapies are being developed with a reasonable safety profile.

RTX is a human/murine chimeric glycosylated immunoglobulin containing murine light- and heavy-chain variable region sequences, and human kappa and human IgG1 constant region sequences. It has specific affinity for the B-lymphocyte transmembrane protein, CD20, which is expressed on normal B-cells (excluding stem cells, pro-B cells, and plasma B cells)^6^. Rituximab infusions elicit circulating and tissue-resident CD20+ cell lysis, but not the destruction of stem or plasma cells^7^. Depletion of memory B cells causes a change in the immune response, with decrease in antibodies and cytokines production and alteration of the process of antigen presentation^8^. In addition to its immunosuppressive role, recent research has demonstrated that the podocyte cytoskeleton may be a direct target for RTX, through modulation of IL17 production and actin cytoskeleton stabilization by the connection with sphingomyelin phosphodiesterase acid-like 3b, leading to podocyte apoptosis^9^^,^ 10.

Generally, B-cell depletion is sustained for up to 12 months in peripheral blood, after which a new B-cell population appears, but it may remain absent for up to 30 months^11^^,^ 12. In recurrent B-cell lymphoma, B cells depletion persists for at least 2-3 months and in glomerular diseases, for 3 to 6 months, but studies showed variable results^13^. This difference is explaining by marked inter-individual variability, and different renal diseases with many factors that influence pharmacokinetics^13^. As RTX is an antibody, patients with nephrotic range proteinuria result in a shortened half-life of RTX that can influence the recovery of B cells^14^.

On the other hand, the molecule is not eliminated by hemodialysis or peritoneal dialysis, but plasma exchange causes the elimination of circulating therapeutic antibodies. Nowadays, there is no generally accepted standard schedule for RTX administration in renal diseases: B-cell driving therapy, conventional therapy dose, or administration driving by ANCAs or PLA2R1 according to etiology of renal disease.

The pharmacokinetic and pharmacodynamic profile of RTX, in a variety of renal diseases, seems to be different. It has been identified using data from clinical studies, but data are weak and rare. Therefore, the real characteristics in these diseases are not well established.

The purpose of this paper is to review the current status of clinical use of RTX in adult patients with idiopathic glomerulopathies.

## Methods

This paper is a narrative review. The authors searched Pubmed-indexed journal using keywords solely or in combination as “ANCA associated renal vasculitis”, “maintenance remission”, “active lupus nephritis”, “refractory lupus nephritis”, “membranous lupus nephritis”, “membranous nephropathy”, “focal segmental glomerulosclerosis”, “minimal change disease”, “membranoproliferative glomerulonephritis”, “immunoglobulin A nephropathy, “Henoch-Schönlein Purpura”, “anti-glomerular basement membrane disease”, “primary glomerulonephritis”, “rituximab”, and “anti-CD20” to find studies evaluating the efficacy of RTX treatment for remission induction or maintenance therapy in adult glomerulopathies. The search was limited to the last 16 years to get the most recent findings of this topic. The studies were eligible according to importance and design. Authors, wherever possible, reported the outcomes of randomized controlled trials (RCTs), followed by prospective and retrospective studies, and case reports. They searched the clinical trial registry also for ongoing trials in “ClinicalTrials.gov” related to the aim of this review.

Articles including pediatric age population, nephrotic syndrome not biopsy-proven, RTX use for glomerular disease treatment in kidney transplantation, use of other monoclonal antibodies than RTX, and articles written in other languages than English, Spanish, or Portuguese were excluded. Authors included only publications about the idiopathic disease in adult native kidneys.

The quality of the included studies was assessed using the Grading of Recommendations Assessment, Development and Evaluation (GRADE) approach^15^.

## Discussion

### Antineutrophil Cytoplasm Antibody Associated Vasculitis

B cells are thought to play an essential role in the pathogenesis of AAV. Not only are B cells the precursors of ANCA secreting plasma cells, but they also act as antigen presenting cells for auto-reactive T cells, providing co-stimulatory support and T cells activation^16^.

Two RCTs, RITUXVAS^17^ and RAVE^18^, demonstrated similar efficacy and safety between RTX and cyclophosphamide for remission induction of AAV. Therefore, the 2012 KDIGO guideline recommended the use of weekly RTX (375mg/m^2^ x 4) as an alternative initial treatment to cyclophosphamide and to treat patients with severe relapse of AAV^19^. However, these guidelines do not make any consideration about RTX as maintenance therapy. Therefore, in this review, the authors reported only the RTX for this purpose.

Recently, non-comparative studies have suggested that RTX with low glucocorticoids dose can be valuable for maintaining remission^20^.

MAINRITSAN was the first RCT that compared the efficacy of RTX (500 mg on days 0 and 14, then at 6, 12, and 18 months) and azathioprine during 22 months in AAV remission maintenance after remission induction protocol using cyclophosphamide. At month 28, the number of relapses was significantly lower in patients administered RTX than in those who received azathioprine (5% vs 29%; *p*=0.002)[21]. On the other hand, MAINRITSAN 2 showed that remissions did not differ significantly between fixed-schedule or individually tailored schedule based on CD19+B lymphocytes counter or ANCA titer, although in this last scheme patients received fewer RTX infusions^22^.

The first trial shows the superiority of RTX over azathioprine in short-term in patients who had achieved remission after cyclophosphamide induction^23^. However, the study was underpowered to assess effective maintenance therapy following remission induction with RTX. The ongoing trial RITIZAREM (NCT 01697267) will attempt to answer this question and will find out whether repeating RTX (1000 mg at 4-month intervals during 20 months) stops vasculitis recurrence and the safety of this scheme.

However, in spite of good results, these two trials were not blinded and patients showed mainly granulomatosis with polyangiitis, which limits the generalizability of the findings. On the other hand, long-term follow-up data have shown high relapse rates in AAV with withdrawal and discontinuation of maintenance agents^24^.

The ongoing trials MAINTANCAVAS (NCT 02749292) and MAINRITSAN 3 (NCT 0243352) study the ideal RTX maintenance therapy scheme concerning fewer relapse rates and toxicity. These studies compare three RTX regimens: infusion based on ANCA titer, CD19 lymphocytes count, or regular infusions.

RTX seems to be better than azathioprine in maintaining remission, as reported by MAINRITSAN trial and evidence-based medicine, suggesting the use of RTX for remission maintenance when combined with low-dose glucocorticoids for 24 months following remission induction. However, the best maintenance schedule and therapeutic duration remain to be defined^25^. In some patients, AAV appears to behave as a chronic and relapsing disease, requiring continued maintenance regimens.

### Lupus nephritis

Systemic lupus erythematosus (SLE) has been associated with several pathogenic autoantibodies directed towards cytoplasm and nuclear cell compartments. There are subsequent immune complex deposition, complement activation, inflammation, and end-organ damage^26^. Dendritic cells, T helper cells, and plasma cells also contribute to the aberrant polyclonal autoimmunity^27^.

The 2012 KDIGO guideline suggested considering RTX to patients with class III/IV LN refractory to conventional therapy^19^. The evidence comes from a small, open-label study, where the addition of 1 g RTX plus 500 mg of intravenous methylprednisolone on days 1 and 15 to conventional immunosuppressive therapy have permitted the achievement of 5/13 complete and 5/13 partial remission^28^. More recently, RTX in the same doses achieved a complete and partial response in 11/18 and 2/18 patients with refractory class III and IV LN, respectively^29^. These studies reinforce the guideline recommendations^30^.

Despite the RTX role in refractory LN, the LUNAR study did not show any benefit of added RTX to initial therapy in 1-year clinical outcomes of patients with active proliferative LN^31^. Although anti-B-cell therapies will eventually decrease inflammation by abrogating the generation of immune complexes, the failure of the above trials indicate that these drugs might be unuseful for induction of remission in patients with LN^32^.

Researchers believe that an association of different targeted drugs, as well as identification of lesions known as being critical for disease pathogenesis (crescents, podocyte injury, tubulointerstitial lesion, renal vascular lesions) could be the solution to induction of lupus nephritis remission^32^^,^ 33.

On the other hand, the LUNAR study did not include patients with pure membranous lupus nephritis^31^. A retrospective study with 15 patients with pure lupus nephritis class V showed remission in 87% of patients treated with RTX as monotherapy (8 patients had complete remission)^34^. Membranous lupus nephritis is caused by deposition of immune complexes in the subepithelial compartments of the glomerular tuft, which ultimately leads to podocyte injury. RTX may be useful because this drug also promotes podocyte stability^32^. However, RCTs in larger cohorts are lacking.

### Idiopathic Membranous Nephropathy (IMN)

The finding of autoantibodies against M-type phospholipase A2 receptor 1 (PLA2R1) and thrombospondin type-1 domain-containing 7A (THSD7A) in serum and their contribution to glomerular immune complexes in IMN patients was a great improvement in our knowledge^35^^,^ 36. Immune complexes cause activation of complements and multiple reactions that lead to basement membrane lesions^37^. The presence of these antibodies provides a clear rationale for the use of anti-B cell therapy.

Uncontrolled trials have reported the efficacy of RTX in IMN patients considered for treatment with immunosuppressive agents, in accordance to KDIGO guideline^19^. Fervenza and Rugenneti et al.^38^^,^ 39 found remission in 60% of patients. However, given the absence of a control group, it is possible that the beneficial effect observed was due to spontaneous remission rather than a therapeutic effect of rituximab. As can be seen in Table 1, other studies showed the efficacy of RTX in IMN treatment^14^^,^ 40. Thus, the RTX best dose is still unknown because different dosing schedules have been used, ranging from one single dose of 1g to 4 weekly dosages of 375 mg/m^2^ 14 ^,^ 38 ^-^ 40.

**Table 1:** Overview the studies included about idiopathic membranous nephropathy

Reference	Disease	Patients (n)	Treatment before RXT	Prot prior RTX g/dia	Cr before RTX, mg/dL	Patients failed previous IMS	RTX dose	Follow-up (mo)	Remission (definition per study)	Cr after RTX, mg/dL	Relapses after RTX	Study design
Remuzzi et al, 2002^40^	IMN	8	Supportive treatment	8.6±1.5	1.4±0.3	0	4 weekly doses of 375mg/m^2^ (5)	5	CR (2), PR (3)	0.79±0.3	NA	Prosp
Fervenza FC, et al, 2008^39^	IMN	15	CyA (7), MMF (7), Csa (7), steroids (7)	13.0±5.7	1.4±0.5	7	1000mg on day 1 and 15 (5)1000mg on day 1 and 15, 6 months apart (10)	12	Cr (2) PR (6); NR (6)	1.6±1	ND	Prosp
RuggenentiP. et al, 2012^38^	IMN	100	Steroids, CyA, Csa, ACTH	5.8-12.8	0.97-1.2	100	4 weekly doses of 375mg/m2 and second infusion if > 5 circulating B cells per mm^3^	29	CR (27); PR (38); NR (35)	1.01-1.6	ND	Prosp
Fervenza FC et al, 2010^14^	IMN	20	Csa (11), CyA (11), MMF (11), TAC (11), steroids (11)	11.9±4.9	1.5±0.5	11	4 weekly doses of 375mg/m^2^, 6 months apart	24	CR (4;) PR(12); NR (1)	1.3±0.3	1	Prosp
Dahan K, et al 2017 GEMRITUX TRIAL)^2^	IMN	75	Supportive treatment at least 6 months	7.4 (4.7-9.7)	1.1 (0.87-1.39)	75	Supportive treatment plus RTX 375mg/m^2^ on days 1 and 8 (37) vs Only supportive treatment (38)	17	CR and PR 24/37 (64.9%) vs 13/38 (34.2%)	1.14 (0.98-1.53)	ND	RCT
van den Brand, et al, 2017^42^	IMN	203	32 patients vs 12 patients received IMS therapy ND	6.4 (4.4-8.9) vs 8.8 (5.7-11.7)	1.2 (0.97-1.7) vs 1.26 (1.0-1.6)	44	4 weekly doses of 375mg/m^2^ and reinfusion if Bcells > 5/mm3 vs Cya 1.5mg/Kg/day, 6-12 mo + MPL +0.5mg/kg oral prednisolone	40	CR (34) vs (26); PR (30) vs (63)	ND	ND	Retro
Fervenza FC et al^41^	IMN	130	Supportive treatment at least 3 months	8.9 (6.8-12.3) vs 8.9 (6.7-12.9)	1.3±0.4 vs 1.3±0.4	130	1000mg on day 1 and 15 and second course if proteinuria reduction ≥25% on 6 months. If CR or NR no second course	24	CR (23), PR (16), NR (26) vs CR (0), PR (13), NR (52)	ND	ND	RCT

ACTH=adrenocorticotrophic hormone; CR=complete remission; Cr=creatinine; CyA= cyclophosphamide; Csa= cyclosporine; IMN= idiopathic membranous nephropathy; IMS= Immunosuppressive therapy; MMF=mycofenolate mofetil; mo=months; NA=not applicable; ND=not defined; NR= no remission; PR=partial remissions; Prot= proteinuria; Prosp= prospective; RCT= randomized controlled trial; Retro= retrospective; RTX=rituximab; TAC= tacrolimus.

GEMRITUX was the first trial that compared non-immunosuppressive antiproteinuric treatment (NIAT) alone or in addition to RTX 375 mg/m^2^ on days 1 and 8. At 24 months (extended and observational period study), significantly more patients in NIAT-RTX group achieved remission when compared to NIAT group, 64.9% vs 34.2% respectively, *p*<0.01^2^. This difference was reported only during the observational period that did not provide the same evidence and, in addition, only seven patients achieved complete remissions in NIAT-RTX group. These results limit the generalizability of the findings because, as it is well known, relapses of nephrotic syndrome and disease progression are more frequent in patients with partial remission.

In the MENTOR trial, the researchers randomized 130 patients with IMN, proteinuria ≥5g/24h and eGFR≥40/mL/min/1.73m^2^, into a 12-month treatment period. Patients received 1000 mg RTX (2 infusions 14 days apart, and reinfusion at 6 months) or 3.5-5mg/kg/day cyclosporine. The results showed complete or partial remission after 24 months in 60% patients of the RTX cohort (35% complete remission) versus 20% partial remission in cyclosporine cohort without any complete remission. However, at 12 months the results were comparable in two cohorts.^41^ Patients seem to have an effective but slow response to RTX. Similarly, van den Brand et al.^42^, in a retrospective study, showed that complete remission rates did not differ between steroid plus cyclical cyclophosphamide (ST-CP) group and RTX group. However, the study design was weak, with patients treated with other kinds of immunosuppression before the study and different inclusion criteria between groups. In this study, 36% of patients on the RTX group did not respond to treatment (the same result achieved in GEMRITUX)^43^. The RCT that compares RTX with ST-CP is ongoing (NCT03018535).

In conclusion, RTX can be an effective alternative in the management of IMN in induction remission. The data indicate that selective B-cell depletion by RTX can be at least as effective as other conventional immunosuppressive regimens^44^. The authors believe that it can be a good option for patients of child-bearing age or patients who have a great cumulative dose of cyclophosphamide, but also as first line therapy, although the efficacy would have to be confirmed. More RCTs will be performed to further evaluate the long-term outcomes, predictable treatment response to RTX, as well as indications as first or second-line therapy regimens.

### Idiopathic focal segmental glomerulosclerosis in adults

The pathophysiology of FSGS remains poorly understood. In FSGS, the podocyte is now viewed as the principal target and, in a subset of patients, a circulating permeability factor may be present. Nevertheless, some studies with children and adults with idiopathic nephrotic syndrome suggest that MCD and FSGS reflect a disease spectrum, as different morphologic manifestations of overlapping etiologic factors^45^.

Nowadays, glucocorticoids are the drugs of choice^19^. In steroid resistant, steroid-dependent or steroid-contraindicated patients, other immunosuppressive therapies are suggested^46^. Adverse effects of the long-term use of such toxic and “non-specific” therapies have led to a demand for more selective immunomodulating and immunosuppressive regimens^47^^,^ 48.

In FSGS, studies using RTX in adults are rare, controversial, and heterogeneous (Table 2). Fernandez-Fresnedo and others have shown that RTX in patients with the steroid-resistant nephrotic syndrome (SRNS) do not have any benefit^49^^-^ 51.

**Table 2:** Overview the studies included about focal segmental glomerulosclerosis and = minimal change disease

Reference	Disease	Patients (n)	Treatment before RXT	Prot. prior RTX g/dia	Cr before RTX, mg/dL	Relapses before RTX	RTX dose	Follow-up (mo)	Remission (definition per study)	Cr after RTX, mg/dL	Relapses after RTX	Treatment after RTX	Study design
Fernandez-Fresnedo et al, 2009^49^	FSGS (SRNS)	8	TAC (3), MMF (7), CyA (2), Csa (7) Steroids (8)	14.0 ± 4.4	1.4 ± 0.5	ND	4 weekly doses of 375mg/m^2^ (5) 4 weekly doses of 375mg/m^2^ followed by the same scheme at 12 mo (1) 4 weekly doses of 375mg/m^2^ followed by the same scheme at 6 mo (1) 8 weekly doses of 375mg/m^2^ (5)	12-24	PR (1) NR (7)	2.2 ± 1.8	ND	ND	Retro
Kong W.Y. et al, 2013^50^	FSGS	4	CyA (2), Csa (2), Aza (1) MMF (1), steroids (3)	ND	ND	12 relapses (1) 0 relapses (3)	375mg/m^2^, single dose	11-51	CR (2), PR (1), NR (1)	ND	1 relapses (1) 0 relapses (3)	Steroids(2)	Retro
Ochi A. et al. 2012^51^	FSGS (2SRNS, 2SDNS)	4	Csa (2), MZ (1), CyA (1), MMF (1), steroids (4),	3.6-13.0	0.5-2.1	2 patients relapses more than 3 times	375mg/m^2^, single dose	variable	CR(2) NR (2)	DN	2 patients relapse	Steroids (2)	Retro
Rocatello D. et al, 2017^52^	FSGS	8	Supportive treatment	5.3 ± 1.9	2.6 ± 1.2	NA	8 weekly doses of 375mg/m^2^	29.1 ± 8.8	NR (7) PR (1)	3.5 ± 2.5	ND	Only supportive treatment	Prosp
Munyentwali et al, 2013^56^	MCD steroid-dependent (15); MCD steroid-resistant (2)	17	TAC(2), MMF (12), Csa (13), CyA (4), LEV (7) mechlorethamina (3), chlorambucil (1), pefloxacin (1), basiliximab (1), steroids (17)	0.05-6.2	ND	All patients had at least 2 relapses	375mg/m^2^, single dose (1) 2 weekly doses of 375mg/m^2^ (7) 3 weekly doses of 375mg/m^2^ (4) 4 weekly doses 375mg/m^2^ (3) 1000mg on day 1 and 15 (2)	5.1-82.2	CR (15), NR (2)	NA	6 patients with at least 1 relapse each	CyA (1); Steroids (4); TAC (1)	Retro
Takei T. et al, 2013^57^	MCD steroid-dependent and frequently relapse	25	MMF (3), CyA (20), MZ (5), steroids (25)	2.5 ± 3.5	0.7 ± 0.2	62 relapses (patients experienced at least 1)	Single dose of 375mg/m^2^,6 months apart, during 1 year (25)	12	CR (25)	0.6 ± 0.1	4 patients with 1 relapse each	CyA (6), steroids (4)	Prosp
Iwabuchi Y. et al, 2014^58^	MCD steroid-dependent and frequently relapse	25	MMF(1), CyA(20), MZ(6), TAC(1)steroids (28)	2.5 ± 4.9	0.7 ± 0.2	108 relapses (patients experienced at least 1)	Single dose of 375mg/m^2^, 6 months apart, during 2 years	24	CR (25)	0.7 ± 0.1	8 patients with 1 relapse each	CyA (5), Steroids (4)	Prosp
Papakrivopoulou E. et al, 2016^59^	MCD steroid-dependent and frequently relapse	15	MMF(3), CyA(5), LEV (3),Csa (6), TAC (4)	ND	0.95 ± 0.3	39 relapses (patients experienced at least 1)	Single dose of 375mg/m^2^, 6 months apart, during 1 year (15)	43	ND	ND	7 patients with 1 relapse each	Dose reduction of TAC and Csa; steroids (0)	Prosp
Fenoglio R. et al, 2018^60^	MCD	6	RTX as first line therapy	11.75 ± 7.8	1.95 ± 1.5	NA	4 weekly doses of 375mg/m^2^	8-36 months	CR (5)	0.86 ± 0.18	0 relapses	None	Case series

Aza= azathioprine; Cr=creatinine; CR=complete remission; CyA= cyclophosphamide; Csa= cyclosporine; FSGS=focal segmental glomerulosclerosis; IMS= Immunosuppressive therapy; LEV= lavamisole; MCD= minimal change disease; MMF=mycofenolate mofetil; mo=months; MZ= mizoribine; NA=not applicable; ND=not defined; NR= no remission; PR=partial remissions; Prosp= prospective; Retro= retrospective; RTX=rituximab; SDNS=steroid-dependent nephrotic syndrome; SRNS=steroid-resistant nephrotic syndrome; TAC= tacrolimus.

In a case series, four patients received RTX. The drug was effective in two cases of steroid-dependent nephrotic syndrome (SDNS) with patients achieving complete remission after two weeks of a single dose of RTX (375mg/m^2^), but not in two patients with SRNS. This study did not report the follow-up period after RTX infusion and patients had different variants of FSGS, global sclerosis, and renal insufficiency^51^. In another similar study, two patients achieved complete remission, one had partial remission, and another did not have any response to RTX. In addition, RTX provided a decrease of additional immunosuppressive medication and a significant relapse reduction. In this study, it was not specified which patients had SRNS or SDNS. Besides, the authors provided their own definitions of remission^50^.

Fernandez-Fresnedo included only patients with nephrotic syndrome resistant to corticosteroids and other immunosuppressive treatments. They achieved partial remission only in one patient, but this effect was transitory. On the other hand, CD20+ lymphocyte counts were undetectable in all patients after RTX administration^49^. This study did not support the effectiveness of RTX treatment in SRNS.

Recently, in a prospective study, RTX (8 weekly doses of 375 mg/m^2^) was used in 8 naïve patients, as first-line treatment. Only one patient had a reduction in proteinuria levels (7 to 1.5 g/24h), without difference in the CD20+ B lymphocyte profile observed in patients with negative response^52^. This cohort had some peculiarities because patients were of high risk for corticosteroids use (diabetes, high BP), which might have resulted in substantial heterogeneity in biopsy findings.

There is an ongoing RCT which compares prednisone to RTX (375mg/m^2^ at time 0 and 14 and repeat dose if no complete depletion of B-cells), in patients unresponsive to 8 weeks of high dose prednisone (NCT03298698).

In conclusion, in the above studies, RTX dose was heterogeneous, even within the same cohort, which makes the comparison of outcomes impossible. A role of RTX in a cohort of idiopathic SDNS FSGS cannot be ignored. In this cohort, RTX might have been useful in reducing the number of relapses and sparing immunosuppression. Different results suggest that not all the pathogenic pathways were identified and reinforce the potential effects of different variants. The reasons for RTX being effective in pediatric patients and transplant recipient while failing to induce improvement in adults with idiopathic FSGS remain unknown. However, in adults, no conclusion can be drawn because studies are scarce and heterogeneous.

### Minimal-change disease in adults

In minimal-change disease (MCD), some patients become steroid-resistant (SR), steroid-dependent (SD) or frequently relapsing (FR)^53^. In more than 50% of adults, the disease will relapse, and one third of them will become frequent relapsers^19^. Historically, MCD has been considered a T lymphocyte pathology. However, recent advances have shown a more complex pathogenesis with the participation of innate immunity, B-cells, regulatory T-cells and circulating factors^54^^,^ 55.

The use of RTX in adults with frequent relapse and immunosuppression-dependent MCD has been reported in prospective and retrospective studies, case reports, and small uncontrolled case series with some success (Table 2).

In one retrospective case series, 17 patients with SD or FR received RTX in different doses. Eleven patients (65%) achieved a sustained complete response with no relapse after a mean of two years of follow up, and nine of them were able to come off all other immunosuppressive drugs and steroids^56^.

In a prospective trial involving 25 patients with SD, after one RTX infusion (375mg/m^2^), only 4 in 25 patients relapsed^57^. Another trial reported 8 relapses after RTX compared to 108 relapses before RTX, in 24 months of follow-up^58^. In this study, all patients achieved complete remission in 24 months, although 20 patients continued with RTX every 6 months, in a single-dose infusion^58^. Recently in another prospective study, two doses of RTX (1g at an interval of 6 months), was effective at maintaining prolonged steroid free remissions and reducing relapse frequency (39 relapses in the year prior RTX vs 7 relapses after RTX, 5 of which occurred when CD19 counts were greater than 100 cells/µL)^59^.

In these studies, patients receiving RTX were not in the same stage of disease (partial remission vs nephrotic syndrome) and the mode of tapering immunosuppressive drugs was not very well defined. However, in all studies, RTX was used to spare steroids and immunosuppressive therapeutics with favorable outcomes. Studies showed mixed results about predicting relapse by the detection of B cells in peripheral blood. Takei et al.^50^ reported relapse always correlated with B-cell repletion, but other studies, as Iwabuchi et al.^51^, documented patients with increased peripheral blood B cell count without recurrence. Papakrivopoulou et al.^52^ and Munyentwali et al.^49^ reported two relapses with CD19 counts below 100 cell/µL. Iwabuchi et al.^58^ reported complete remissions during at least 12 months after 5 RTX infusions. However, the follow-up period of other studies was relatively short.

On the other hand, in naive patients, RTX (4 weekly doses of 375 mg/m^2^) as first-line therapy without the association of steroid/immunosuppressive drugs showed sustained complete renal remission in 5/6 patients, while in one patient proteinuria decreased 75%. All patients had nephrotic syndrome and none relapsed during the follow-up of 8-36 months^60^ despite the recovery of B-cell count.

Although RTX appears to be effective as first-line therapy or a steroid/immunosuppressive-sparing drug in MCD, no RCTs in adults have been conducted comparing RTX treatment alone to other currently used agents such as cyclophosphamide, cyclosporine, and MMF. The causes of protracted remission in some patients or relapse in other patients have not yet been clarified, as well as the relationship between relapse and B-cell count.

### Idiopathic Membranoproliferative Glomerulonephritis (MPGN)

MPGN was historically classified into three categories: type I, type II, and type III, based on electron microscopy findings^61^. With an improved understanding of its pathogenesis, Sethi and Fervenza proposed a new classification of MPGN into two major groups^62^.

### Immunoglobulin-mediated MPGN

Most cases of immunoglobulin-mediated MPGN are secondary to infections, autoimmune disease, and monoclonal gammopathies. The diagnosis of idiopathic MPGN is established after all of these secondary causes are excluded. Therefore, idiopathic MPGN is decreasing in frequency^63^. There are in literature very few studies of RTX in this condition.

A retrospective study (Table 3) about RTX treatment in primary glomerulonephritis included two patients with idiopathic MPGN. The patient who received RTX in a single dose of 500 mg achieved complete remission after 19 months and the patient who received RTX 800 mg in two doses reached partial remission 29 months later^50^. The aim of that study was to explore the efficacy and safety of RTX therapy in adults with primary glomerulonephritis. Therefore, it is hard to rush into a conclusion about a specific disease. In an open-label trial, there was a reduction of proteinuria in six patients with MPGN type I treated with RTX (1 g on day 1 and day 15) after 6 months: 2 patients had complete remissions and 3 patients had partial remissions^64^. As shown above, RTX appeared to be effective. Once this disease tends to follow a progressive course without spontaneous remissions, in this trial the absence of a control group would not be relevant. Thus, remissions were likely treatment-related mainly because patients did not make another IMS therapy. However, a larger trial would be essential as well as a trial that compares RTX to other IMS therapies. Currently, there is an ongoing RCT (NCT03180723) that compares RTX to cyclosporine as first-line therapy.

**Table 3:** Overview the studies included about membranoproliferative glomerulonephritis

Reference	Disease	Patients (n)	Treatment before RXT	Prot. prior RTX g/dia	Cr before RTX, mg/dL	Relapses before RTX	RTX dose	Follow-up (mo)	Remission (definition per study) or prot (g/day)	Cr after RTX, mg/dL	Relapses after RTX	Treatment after RTX	Study design
Dillon JJ et al, 2012^64^	IG MPGN	6	Supportive treatment and steroids	3.9 ± 2.0	1.7 ± 0.5	1000mg on day 1 and 15	1000mg on day 1 and 15	12	CR 2;PR 3; NR 1	1.7	1	none	Prosp
Giaime P et al., 2015^67^	C MPGN	1	Supportive treatment	6	2.7	ND	4 weekly doses of 375mg/m^2^, 18 months apart	30	< 0.5	ND	ND	Supportive treatment	Retro
Kong W.Y. et al, 2013^50^	IG MPGN	2	none	none	ND	ND	Single dose of 500mg (1) 2 doses of doses of 800mg, periodicity unknown (1)	43-32	CR (1) PR (1)	ND	0	Steroids (1)	Retro

CR=complete remission; Cr=creatinine; C MPGN= complement-mediated membranoproliferative glomerulonephritis; IG MPGN= Immunoglobulin-mediated membranoproliferative glomerulonephritis; C G IMS= Immunosuppressive therapy; mo=months; ND=not defined; NR= no remission; PR=partial remissions; Prot= proteinuria; Prosp=prospective; Retro= retrospective; RTX=rituximab.

### Complement-mediated MPGN

Complement-mediated MPGN is characterized by defects in the alternative pathway of complement, producing C3 immunoglobulin deposition. This group includes dense deposit disease (DDD) and C3 glomerulonephritis (C3GN)^65^. B-cell depletion is beneficial when autoantibodies cause the disease, such as the case with C3 nephritic factor (C3NeF) or autoantibodies against inhibitory proteins of the alternative pathway (factor H, I, MCP) leading to an uncontrolled activation of the complement cascade^66^.

One patient with C3NeF treated with 700 mg RTX weekly for a month as the sole immunosuppression regimen obtained a sustained complete remission in 6 months^67^. The other published case reports were on children. One study is not enough to get the right conclusions about treatment efficiency. It is natural to think that RTX works against autoantibodies such as C3Nefs, which leads to an uncontrolled activation of the complement cascade^68^. Therefore, in the case mentioned above, C3Nef remained positive during the follow-up period. At least in this patient, the exact role of C3Nef and complement system is questionable, as is, consequently, the real mechanism of RTX (a immune phenomenon or only a nephroprotective agent).

There is a great paucity of data and therefore it is not possible to draw meaningful conclusions. Further studies will be critical to clarify the RTX role in this pathology. On the other hand, eculizumab inhibits activation of terminal complement complex and may provide a better target therapy in complement-mediated GNMP^69^.

### Immunoglobulin a nephropathy

Recent studies have confirmed the autoimmune nature of IgAN and suggested a multihit pathway influenced by genetic factors, in which galactose-deficient polymeric IgA1 is identified by autoantibodies that consequently drive the formation of nephritogenic immune complexes^70^. IgAN pathogenesis develops in 4 steps not completely understood^71^. For instance, mucosal infection is now appreciated as strongly correlated with a higher risk of developing IgA nephropathy^72^.

Aggressive immunosuppression is reserved only to crescentic IgAN with rapid deterioration of kidney function^19^. There is a lack of studies reporting RTX treatment in IgAN (Table 4). A case series reported two adults patients treated with RTX with crescentic IgAN. They were treated with methylprednisolone pulses, followed by prednisone daily and 1 g RTX at an interval of two weeks. In one patient with 31% crescents, creatinine decreased from 2.64 to 0.88 mg/dL and urine albumin:creatinine ratio (ACR) decreased from 1415.93 to 77.9 mg/g. In addition, in the patient with 80% crescents, creatinine decreased from 4.0 to 2.1 mg/dL and ACR decreased from 3867.3 to 221.24 mg/g after one year^73^. On the other hand, in a 68-year-old woman with purpura nephritis associated with nephrotic syndrome, treatment with RTX (375 mg/m^2^ weekly for 4 weeks) as well as steroids achieved complete remission in four weeks^74^. Similar results have been found in another case report^75^. In the last two studies, the biopsy did not show crescents in more than 50% of glomeruli and patients did not present with rapidly progressive renal deterioration. According to KDIGO guidelines, it is not clear the intention to treat with IMS therapy in these cases. An initial course of corticosteroids seemed enough in the study by Pillebout et al.^75^ however, Ishiguro et al.^74^ used RTX as the second line following failure of steroids and cyclophosphamide, with excellent outcomes.

**Table 4:** Overview the studies included about Immunoglobulin A nephropathy

Reference	Disease	Patients (n)	Treatment before RXT	PCR/ACR or Prot prior RTX g/gCr or g/day	Cr before RTX, mg/dL	RTX dose	Follow-up (mo)	PCR/ACR or Prot after RTX g/gCr or g/day	Cr after RTX, mg/dL	Treatment after RTX	Study design
Lundberg S. et al, 2017^73^	Crescentic IgAN	2	MPN pulses, prednisolone 0.6mg/Kg/day	ACR:1.73-2.8	1.24-2.35	1000mg on day 1 and 15	20-17	ACR: 0.08-0.7	0.68-2.22	MMF (1)	Retro
Ishiguro H. et al, 2013^74^	Crescentic IgAVN	1	MPN pulses, prednisolone 0.6mg/Kg/day; Cya;	Prot: 6.6	0.58	4 weekly doses of 375mg/m^2^	12	Prot.≈0	ND	none	Retro
Pillebout E. et al, 2011^75^	Crescentic IgAVN	4	Supportive treatment	Prot:1.1	1.1	1000mg on day 1 and 15	22	Prot.≈0	0.85	none	Retro
Sugiura H. et al, 2011^76^	IgAN	5	Steroids (4)	1.0±0.8	1.1±0.3	Single dose of 375mg/m^2^,	6	0.9±0.8	1.1±0.3	Steroids (2)	Retro
Lafayette RA et al, 2017^77^	IgAN	34 (17 patients each group)	Steroids (11), 6 in RTX group and 5 in other group	2.1 (0.6-5.3)	1.4 (0.8-2.4)	1000mg on day 1 and 15, 6 months apart vs supportive treatment	12	1.9 (0.4-8.8) vs 1.8 (0.4-4.3)	1.7 (0.8-2.3) vs 1.3 (0.8-2.4)	Supportive treatment	RCT

ACR= Albumin creatinine ratio; Cr=creatinine; IgAN=Immunoglobulin A nephropathy; IgAVN= IgA vasculitis with nephritis; IMS= Immunosuppressive therapy; MMF=Mycophenolate mofetil; mo=months; MPN=Methylprednisolone; ND=not defined; PCR= Protein creatinine ratio; Prot= proteinuria; RCT= randomized controlled trial; Retro= retrospective; RTX=rituximab.

Furthermore, in 5 patients with IgAN treated with RTX in a single-dose of 375 mg/m^2^ there was no significant change in proteinuria at 6 months of follow up, while depletion of CD19 and CD20 cells was noted^76^. A recent RCT, involving 34 adult patients with biopsy-proven IgA nephropathy with < 50% glomerular sclerosis or interstitial fibrosis and proteinuria > 1g/d, showed that RTX (1 g at an interval of two weeks) did not significantly improve renal function or proteinuria and severity of disease in this stage^77^. This study also failed to reduce serum levels of galactose-deficient IgA 1 and anti-galactose-deficient IgA1 antibodies, assigning salient pathogenic roles in IgA nephropathy.

Increasing data suggest that immunosuppression is not particularly useful in IgA nephropathy, particularly in early stages or in patients with relatively high risk for progressive renal dysfunction resulting in insignificant reductions in proteinuria that could not be distinguished from the response to supportive therapy alone, as shown in Sugiura et al.^76^ and Lafayette et al.^77^. However, RTX may play an important role in crescentic IgA or Henoch-Schönlein purpura. More studies, including RCTs, will be critical to clarify the role of RTX, and its superiority and safety in comparison to other drugs or supportive therapies in cases of IgAN, crescentic IgAN, or Henoch-Schönlein purpura.

### Anti-glomerular Basement Membrane Antibody Glomerulonephritis

Anti-GBM GN treatment is based on removing the pathogenic antibody that causes the disease, in addition to suppressing the further synthesis of those antibodies^19^. Therefore, RTX appears to play a role in this glomerulonephritis treatment by suppressing the formation of new anti-GBM antibodies.

The KDIGO guidelines recommend initiation immunosuppression with cyclophosphamide and corticosteroids plus plasmapheresis, but suggest studies comparing RTX to cyclophosphamide, both combined with prednisone plus plasmapheresis, for induction of remission^19^.

In a case series of 3 patients, GBM antibodies disappeared after a RTX-based regimen. This study included one patient treated with RTX as first-line therapy. This patient received two doses of 375mg/m^2^ RTX one week apart in addition to methylprednisolone and plasma exchange^78^. GBM antibodies reduced to < 3 U/mL 20 days after initial RTX infusion. There was an exceptional recovery of renal function with 3.1 mg/dL of serum creatinine on admission and 1.13 mg/dL after 33 months. Another similar study showed partial recovery of the renal function and end of dialysis after six weeks of treatment with pulsed methylprednisolone, plasma exchange, and 1 g RTX (on day 5 and day 11)^79^. In this case report, the patient had severe disease with an initial serum creatinine of 20 mg/dL and he required dialysis on admission. This proves that in selected cases, the disease can be successfully treated and RTX can be a reasonable treatment of severe disease.

On the other hand, a retrospective study included four patients with severe disease manifestations. They received four weekly pulses of 375 mg/m^2^ RTX associated with daily plasma exchange as first-line therapy. In the end, RTX was useful to treat pulmonary manifestations, but renal outcomes were not significantly improved^80^. The limited number of patients and the lack of a control group did not allow general conclusions. Similar findings were presented by Narayanan M. et al.^81^.

There are no ongoing RCTs about RTX in anti-GBM disease. Given the rarity of the condition, it is challenging to perform RCTs. The rare findings suggest that RTX effectively induced complete resolution of pulmonary hemorrhage. However, the renal outcome did not significantly improve in dialysis-dependent patients at presentation.

### Safety of Rituximab

Besides RTX efficacy, it is likewise essential to ensure a favorable safety profile over other immunosuppressive drugs used in glomerulopathies treatment. RTX seems safe and well tolerated in most patients. However, several severe adverse events in lymphoproliferative or autoimmune diseases were observed.

The main adverse-events observed in RTX treatment were infusion-related reactions. They occurred almost always during the first RTX administration in 24-36% of patients as cough, hiccups, and exanthema^14^^,^ 38 ^,^ 58. These events recovered with only temporary interruption of the infusion or, in exceptional circumstances, with hydrocortisone. Premedication 30 to 60 minutes before each infusion using 10 mg of clemastine, 1000 mg of acetaminophen, associated with RTX slow infusion rate, with or without corticosteroids, decrease the risk of these events. Mild symptoms as muscle pain, patches of hair loss, hair thinning, fatigue, voice loss, and flu-like symptoms were also reported after RTX infusion^14^^,^ 39.

A reasonable increase in infection risk after RTX has become apparent, although confounding factors are often present^82^. In a retrospective study of 370 RTX patients, the rate of serious infections events was 3.7%, with the majority occurring within the first seven months after RTX initiation^83^.

When the studies above were revised we concluded that severe adverse events were rare. Even in RCTs, patients treated with RTX and other immunosuppressive therapy or supportive therapy did not show any significant differences regarding adverse events, number of infections, cancer, and death rates^2^^,^ 22 ^,^ 31 ^,^ 38. Iwabuchi et al. reported leukopenia at 9 months from the baseline, and studies in patients with lupus nephritis showed an association between leukopenia and RTX treatment^28^^,^ 29 ^,^ 31 ^,^ 58. The infections were uncommon and occurred usually during 2-26 months, mainly lower and high respiratory tract infection, gastroenteritis, and viral herpes zoster reactivation.^34^^,^ 39 ^,^ 49 ^,^ 59.

Some authors reported infections and cardiovascular events clustered in the subgroup of patients who did not achieve remission. A reduction of these events in patients who achieved remission and the increase of adverse events in patients without immunosuppressive therapy, suggests an association with the underlying disease rather than treatment^38^^,^ 43 ^,^ 77. Thus, risks of infection in RTX recipients may depend more on characteristics of patients, disease, and the usually combined glucocorticoid treatment and not only on the cumulative RTX dose^84^^,^ 85.

Severe complications such PML, pneumocystis pneumonia, and fulminant hepatitis have been ascribed to RTX.

One study identified 57 patients with PML from 1997 to 2008, and all of these patients had previously been exposed to corticosteroids and several chemotherapeutic agents without RTX as the only IMS medication^86^. In the above studies, none of the patients presented PML, but due to the high death rate, it is critical to keep in mind the possibility of developing PML. However, at this time there are no recommendations to screen patients for JCV^87^.

Pneumocystis pneumonia (PCP) has been ascribed to RTX but patients also were exposed to other immunosuppressant drugs. In the MAINRITSAN trial, PCP was observed in one patient in the RTX arm^21^. Elsegein et al.^88^ showed that mice treated with anti-CD20 are incapable of mounting a protective immune response to Pneumocystis infection reinforcing the importance of prophylaxis with trimethoprim/sulfamethoxazole^90^^,^ 91.

In 2013, the FDA warned about the risk of reactivation of hepatitis B virus (HBV) infection recommending to test for HBsAg and anti-HBc before treatment initiation. If tests were positive, baseline HBV DNA levels should be measured and consultation with hepatologists regarding antiviral treatment is advised^10^.

Lastly, repeated RTX administration has been reported to reduce immunoglobulin levels but studies did not reveal any new safety issues^91^^,^ 92. However, there are rare reports of patients who developed repeated RTX-induced serum sickness (RISS). Although a rare complication, clinicians should be aware of RISS symptoms and avoid further infusions of RTX to such patients^93^^,^ 94.

The studies above had a too short follow up to achieve the long-term side effects of RTX therapy. The majority of data of severe complications come from patients with autoimmune-disease or lymphoproliferative disorders exposed to extremely high doses of RTX or receiving life-long therapy. These diseases result in a different number of circulating CD20 cells from glomerulopathies and consequently these patients may have an increased risk for adverse events and infusion reactions^95^. However, the studies on glomerulopathies show that RTX is remarkably safe particularly when compared with other immunosuppressants^42^^,^ 96 ^,^ 97. The authors believe that infection risk and other complications can be reduced as RTX allows withdrawal from steroids and other immunosuppressants. However, we should be aware that most studies were designed to evaluate RTX as first therapy or for stopping corticosteroids and not to evaluate the safety profile.

## Conclusion

The management of idiopathic glomerulopathies remains a challenge for nephrologists. Studies are rare, include few patients, and the pathophysiology of these diseases is uncertain and not fully understood.

The evidence for RTX use in glomerular disease is feeble and more RCTs are needed to draw definitive conclusions. In the glomerular diseases reported above, most data came from uncontrolled case series or case reports, and the potential for publication bias, spontaneous remission of disease, and concurrent or previous treatments must be considered.

On the other hand, much of the information regarding RTX safety has been described from hematology and rheumatology experience^10^^,^ 98. Also, in nephrology, RTX showed a small incidence of adverse events and toxicity in many studies^38^^,^ 41 ^,^ 55 ^,^ 98 but others as RAVE and RITUXVAS studies described severe infections in 7% and 18%, respectively, and surprisingly, did not differ from the cyclophosphamide arm^17^^,^ 18. A recent study (n=98) of rituximab use in glomerular disease described an overall infection rate of 21.6 per 100 patient years^99^. Authors admitted that infections rates after RTX therapy vary according to the indication of the treatment, age, comorbidities, and the cumulative effect of other immunosuppressive agents^99^. Other complications as infusion reactions, hypogammaglobulinemia, and late-onset neutropenia can also occur.

In conclusion, current data show a role of RTX mainly in the management of patients with MCD who are steroid-dependent or frequently relapsing and with IMN. On the other hand, the KDIGO guidelines recommended RTX use for AAV induction treatment and in non-responder patients with LN. Recent studies show that RTX also seems to be better than azathioprine in maintaining remission in AAV. More RCTs are needed to establish the real role of this drug in glomerular disease and the risk-benefit of RTX compared to conventional therapy (Table 5).

**Table 5:** Summary of the rituximab use in glomerulopathies, based on current evidence

Rituximab use	AAV	Lupus nephritis	IMN	FSGS	MCD	MPGN	IgAN	Anti-GBM GN
Recommended/ Effective according to current evidence	RTX is not inferior to CP. Therefore, in remission induction, it is recommended as a possible first-line therapy.	RTX is recommended to treat patients with class III/IV LN refractory to conventional therapy.	In induction of remission, RTX appears to be more effective than NIAT and not inferior to cyclosporine or ST-CT.		RTX is effective in patients with MCD who are steroid-dependent or frequently relapsing.			
In study/ more studies are required	RTX seems to be better than azathioprine, as maintenance remission, although RCTs are needed to confirm the best maintenance schedule (fixed schedule or individually tailored) and duration.	RTX may be useful as monotherapy in class V LN (one study showed remission in 87% of patients), but RCTs are needed to confirm this.	RCTs are needed to provide accurate indications for RTX use instead of other IMS drugs, as first or second-line therapy. The dosing schedules and treatment duration must also be determined.	In previous studies, RTX appears to be useful in SDNS, as IMS sparing drug, but in SRNS it seems not to be effective. However, RCTs are needed to clarify indications for RTX use in FSGS.	RCTs are needed to compare RTX to currently used agents. The best dosing schedule (including maintenance therapy) and predictors of relapse also required to be established.	In idiopathic immunoglobulin-mediated MPGN, RTX appears to be effective, but RCTs that compare RTX to other IMS therapy are essential.	In previous studies, RTX seems to play a significant role in crescentic IgA and Henoch-Schönlein purpura. However, RCTs are needed to confirm this, as well as its possible inefficacy in IgAN.	In previous studies, RTX successfully treated dialysis-independent patients and pulmonary manifestations of anti-GBM GN. Studies to evaluate the role of RTX compared to CP are needed.

AAV= Antineutrophil cytoplasm antibody associated vasculitis; anti-GBM GN= Anti-glomerular basement membrane antibody glomerulonephritis; CP= Cyclophosphamide; CR= Complete remissions; FSGS= Idiopathic focal segmental glomerulosclerosis; IgAN= Immunoglobulin A nephropathy; IMN= Idiopathic membranous nephropathy; IMS= Imunossupression; LN= Lupus nephritis; MCD= Minimal change-disease; MPGN= Membranoproliferative glomerulonephritis; NIAT= Nonimunossupressive antiproteinuric treatment; SDNS= Steroid-dependent nephrotic syndrome; SRNS= Steroid-resistant nephrotic syndrome; RCTs= Randomized controlled trials; RTX= Rituximab; ST-CP= Steroid plus cyclical cyclophosphamide.
